# Memory Recall for High Reward Value Items Correlates With Individual Differences in White Matter Pathways Associated With Reward Processing and Fronto-Temporal Communication

**DOI:** 10.3389/fnhum.2018.00241

**Published:** 2018-06-15

**Authors:** Nicco Reggente, Michael S. Cohen, Zhong S. Zheng, Alan D. Castel, Barbara J. Knowlton, Jesse Rissman

**Affiliations:** ^1^Department of Psychology, University of California, Los Angeles, Los Angeles, CA, United States; ^2^Department of Psychology, Northwestern University, Evanston, IL, United States; ^3^Department of Psychiatry & Biobehavioral Sciences, University of California, Los Angeles, Los Angeles, CA, United States

**Keywords:** diffusion tensor imaging (DTI), encoding, semantic, uncinate fasciculus, nucleus accumbens, ventral tegmental area, probabilistic tractography

## Abstract

When given a long list of items to remember, people typically prioritize the memorization of the most valuable items. Prior neuroimaging studies have found that cues denoting the presence of high value items can lead to increased activation of the mesolimbic dopaminergic reward circuit, including the nucleus accumbens (NAcc) and ventral tegmental area (VTA), which in turn results in up-regulation of medial temporal lobe encoding processes and better memory for the high value items. Value cues may also trigger the use of elaborative semantic encoding strategies which depend on interactions between frontal and temporal lobe structures. We used diffusion tensor imaging (DTI) to examine whether individual differences in anatomical connectivity within these circuits are associated with value-induced modulation of memory. DTI data were collected from 19 adults who also participated in an functional magnetic resonanceimaging (fMRI) study involving a value-directed memory task. In this task, subjects encoded words with arbitrarily assigned point values and completed free recall tests after each list, showing improved recall performance for high value items. Motivated by our prior fMRI finding of increased recruitment of left-lateralized semantic network regions during the encoding of high value words (Cohen et al., 2014), we predicted that the robustness of the white matter pathways connecting the ventrolateral prefrontal cortex (VLPFC) with the temporal lobe might be a determinant of recall performance for high value items. We found that the mean fractional anisotropy (FA) of each subject’s left uncinate fasciculus (UF), a fronto-temporal fiber bundle thought to play a critical role in semantic processing, correlated with the mean number of high value, but not low value, words that subjects recalled. Given prior findings on reward-induced modulation of memory, we also used probabilistic tractography to examine the white matter pathway that links the NAcc to the VTA. We found that the number of fibers projecting from left NAcc to VTA was reliably correlated with subjects’ selectivity index, a behavioral measure reflecting the degree to which recall performance was impacted by item value. Together, these findings help to elucidate the neuroanatomical pathways that support verbal memory encoding and its modulation by value.

## Introduction

As we go about our day-to-day lives, we often find ourselves bombarded with new information, only some of which may be important to remember. A growing body of research has begun to characterize the cognitive and neural mechanisms that support our ability to prioritize the encoding of those items that we believe will be most valuable to later recall (for reviews see Castel, 2008; Shohamy and Adcock, 2010; Miendlarzewska et al., 2016). In an experimental setting, the relative importance of individual items is typically conveyed to participants by cues indicating the point value or reward magnitude that could be earned if that item is correctly remembered on an ensuing test. Functional magnetic resonance imaging (fMRI) studies have found that cues denoting the presence of high reward value items can lead to increased activation of the mesolimbic reward circuit, including the nucleus accumbens (NAcc) region of the ventral striatum and the ventral tegmental area (VTA) of the midbrain (Adcock et al., 2006; Cohen et al., 2014). The NAcc, which receives inputs from the ventromedial prefrontal cortex (PFC) conveying information about motivational salience, is thought to represent the magnitude of anticipated reward (Delgado et al., 2000; Knutson et al., 2001). Its projections to dopamine-producing neurons of the VTA can trigger the release of dopamine into the hippocampus, promoting synaptic plasticity via long-term potentiation, which serves to strengthen one’s memory for information encountered in close temporal proximity to the value cue (Lisman and Grace, 2005). While the engagement of these mechanisms may be automatically triggered in response to value cues, such cues may also serve to promote memory encoding by encouraging the individual to allocate increased attention to high value information and employ cognitive strategies to process that information in a more effective manner (Cohen et al., 2017; Middlebrooks et al., 2017). One particularly effective strategy is the engagement of elaborative encoding processes, in which an item’s semantic attributes are processed in a deep manner (Craik and Tulving, 1975; Castel, 2008). This often entails the effortful generation of visual images, associations, or stories in an effort to make the item’s representation more memorable. Recent evidence from fMRI studies indicates that engagement of the brain’s so-called “semantic network” (Binder and Desai, 2011) which includes regions of the left ventrolateral PFC (VLPFC) and lateral temporal cortex, is markedly increased during the encoding of high value items (Cohen et al., 2014, 2016). Although functional neuroimaging studies like these have contributed to our understanding of these two putative mechanisms of reward value-induced memory enhancement—one tied to the brain’s dopaminergic reward circuitry and one tied to strategic engagement of the semantic network—these studies have also highlighted substantial individual differences in the degree to which people engage these mechanisms (Adcock et al., 2006; Cohen et al., 2014, 2016).

In the present study, we sought to examine whether individual differences in the degree to which item reward value impacts memory encoding might be at least partially explained by individual differences in the structural integrity of key anatomical pathways within the brain’s reward system and semantic control system. To accomplish this, we used diffusion tensor imaging (DTI) data to measure the structural characteristics of several white matter pathways that we hypothesized might have relevance to reward value-incentivized remembering. One such pathway of interest was the uncinate fasciculus (UF), a fiber tract that connects portions of the inferior PFC with the anterior temporal lobe (Schmahmann et al., 2007; Von Der Heide et al., 2013; Leng et al., 2016; Hau et al., 2017). Prior DTI studies have strongly implicated the UF in both semantic processing (Matsuo et al., 2008; McDonald et al., 2008; Acosta-Cabronero et al., 2011; de Zubicaray et al., 2011; Galantucci et al., 2011; Agosta et al., 2012) and aspects of episodic memory (Diehl et al., 2008; Lockhart et al., 2012; Thomas et al., 2015; Wendelken et al., 2015; Alm et al., 2016).

Although our primary candidate for a white matter pathway involved in controlled semantic processing and verbal memory was the UF, we also examined the putative role of another major pathway—the inferior frontal occipital fasciculus (IFOF). This pathway connects ventrolateral PFC regions with more posterior areas of the temporal cortex, as well as with some occipital regions (Catani and Thiebaut de Schotten, 2008). Individual differences in the integrity of this pathway have also been linked to behavioral performance on tests of semantic memory (de Zubicaray et al., 2011) and semantic control (Nugiel et al., 2016), and damage to this pathway can lead to semantic paraphasias (Mandonnet et al., 2007).

Finally, with respect to the brain’s reward circuitry, our analysis focused on examining whether the robustness of the connection between the NAcc and VTA (Morales and Margolis, 2017) would be predictive of individual differences in reward value-based modulation of memory. Prior DTI work has associated increased NAcc-VTA connectivity with better reward learning performance (Samanez-Larkin et al., 2012). Furthermore, fMRI-based measurements of functional connectivity have reported strong coupling between NAcc and VTA during the intrinsic resting state (Kahn and Shohamy, 2013), as well as heightened coupling between these regions during novelty-induced reward anticipation (Krebs et al., 2011).

For each of these candidate pathways, we derived metrics of white matter integrity from the DTI data of individual participants, who also performed a value-directed remembering task (Cohen et al., 2014). The task was designed to incentivize selective encoding of valuable information (Castel, 2008). Specifically, on each trial, participants were presented with a high or low value cue that preceded the display of a unique word and indicated the number of points they would earn if they subsequently recalled that word. Given the relatively large number of words on each list, participants were unlikely to remember them all, and thus it was advantageous for them to prioritize the memorization of words associated with a high value in their attempt to maximize their point total. It is important to note that although the points accumulated by participants in this task had no tangible reward value (i.e., they could not be converted to a monetary payout), the motivational salience of these point values was reflected in both participants’ memory behavior and in the value-modulated engagement of reward-related regions in the midbrain and ventral striatum (Cohen et al., 2014). We quantified a participant’s success on this value-directed remembering task using three metrics: the average number of high and low value words recalled per list (Mean High Recall and Mean Low Recall) and “Selectivity Index,” a putative trait variable that indexes the degree to which each participant prioritized the memorization of high value items over low value items (Castel et al., 2002). To the extent that successful recall of high value words depends on the engagement of deep semantic processing during encoding (Cohen et al., 2014, 2017), we hypothesized that participants’ ability to remember high value items would correlate with individual differences in the structural integrity of the UF and/or IFOF pathways that have been implicated in semantic control, and potentially also with the NAcc-VTA pathway associated with reward processing. We furthermore hypothesized that individual differences in the robustness of the NAcc-VTA pathway might correlate with variability in reward-related modulation of learning, as captured by their Selectivity Index measure.

## Materials and Methods

### Participants

Twenty-two adults were enrolled in this study. Data from three participants were excluded from analysis: one for being a non-native English speaker and two for whom we were unable to acquire diffusion-weighted MRI data (one participant’s scanning session was discontinued due to discomfort and the other due to time constraints). The remaining 19 participants (10 female; mean age = 21.8 ± 3.7 years) were all right-handed, native English speakers who reported no current psychoactive medications or severe psychiatric or neurological disorders. All participants either had normal or corrected-to-normal vision. Participants were recruited via flyers placed around the UCLA campus and were remunerated for their participation. Written informed consent was obtained from each participant, and all procedures were approved by UCLA’s Medical Institutional Review Board (IRB #11-002443).

### Behavioral Procedure

A value-directed memory task, adapted from an experimental paradigm developed by Castel et al. (2002) and Castel (2008), was administered in the MRI scanner as participants underwent functional imaging. Extensive details about the protocol have been previously reported (Cohen et al., 2014, 2016), and key elements are summarized below. Participants performed five study-test cycles, each consisting of the study of a list of 24 unique words followed by a free recall test; in addition, two study-test cycles were completed as practice prior to the scanning session. The words were 4–8 letter concrete nouns, and each was assigned a point value indicating how many points could be earned if that word was later recalled. Half of the words were arbitrarily assigned a high value (10, 11, or 12 points) and the other half were assigned a low value (1, 2, or 3 points); value assignment was counterbalanced across participants. During each study list, the presentation of 12 high value and 12 low value words was intermixed in a pseudorandomized fashion. Each trial began with a numeric value cue presented inside of a gold coin symbol (2 s), followed by a fixation cross (3–6.75 s). Then, the to-be-remembered word was presented (3.5 s) followed by another fixation (1.5 s). During the inter-trial interval (3.75–8.75 s), participants performed a simple vowel/consonant judgment task designed to prevent continued rehearsal of the words. Upon the conclusion of each 24-word list, fMRI scanning momentarily ceased and participants were given 90 s to recall as many words as possible from the preceding list, with an emphasis to maximize their total point score. Immediately after recall was complete, participants were given feedback on the points earned for that list.

In order to index the degree to which each participant selectively prioritized the memorization of high value items, while taking into account the overall memory ability of that participant, we computed a measure known as *Selectivity Index* (Castel et al., 2002) using the formula: (actual score − chance score)/(ideal score − chance score), where “actual score” indicates the total number of points earned, “chance score” indicates the point total that the participant would have earned had the point values been randomly assigned (i.e., mean point value multiplied by number of words recalled), and “ideal score” indicates the point total that would have been earned if the *N* words that the participant recalled were only those with the highest *N* point values. As an illustrative example, if a participant recalled only four words on a given list, and the points associated with these words were 12, 10, 11 and 12, then the participant’s Selectivity Index would be very high. The ideal score for four words would be 48 (since there are four 12-point words on each list); the participant’s actual score would be 45; the chance score would be 26, since the average point value across all list items was 6.5 points; thus, the Selectivity Index in this case would be: (45 − 26)/(48 − 26) = 0.86. In this way, we calculated the Selectivity Index for each list, and then averaged across lists to yield a single score.

### MRI Scanning Procedure

MRI data were acquired on a 3.0T Siemens Tim Trio Scanner at the UCLA Staglin IMHRO Center for Cognitive Neuroscience equipped with a 12-channel receive-only phased array head coil. A high-resolution T1-weighted anatomical image was obtained using a 3D MPRAGE sequence (TR = 1900 ms, TE = 3.26 ms, flip angle = 9°, FoV = 250 mm, voxel size = 0.98 × 0.98 × 1.0 mm). Diffusion weighted imaging data were obtained using a multi-directional diffusion weighting (MDDW) spin-echo echoplanar imaging (EPI) sequence (64 non-collinear directions, *b*-value = 1000 s/mm^2^, TR = 9000 ms, TE = 93 ms, echo spacing = 0.69 ms, 60 axial slices, FoV = 190 mm, voxel size = 2.0 × 2.0 × 2.0 mm) with a non-diffusion weighted reference volume (*b* = 0 s/mm^2^). Prior to the acquisition of these structural scans, functional EPI data were obtained as participants performed the value-directed memory task; results from analysis of those data have been previously reported (Cohen et al., 2014). Stimuli were presented using E-Prime 2.0 software (Psychology Software Tools, Pittsburgh, PA, USA), and images were shown via either a custom-built MR-compatible rear projection system, or via MR-compatible goggles (Resonance Technology, Inc.).

### Diffusion Tensor Imaging Data Processing

Diffusion MR data were preprocessed using the FMRIB’s Diffusion Toolbox (FMRIB Software Library, FSL version 5.0.6^1^). All diffusion-weighted images were corrected for eddy currents and aligned to the b0 reference volume. A brain-tissue-only mask was created for each subject using Brain Extraction Tool (BET) and applied to all images. Tensor models were fit to the diffusion data from each voxel using DTIFIT to produce whole-brain fractional anisotropy (FA) maps for each subject.

All analyses were conducted in subject-specific diffusion space in an effort to minimize resampling of the diffusion data. Because our principal analyses involved several regions-of-interest (ROIs) that were defined in standard Montreal Neurological Institute (MNI) template space, these ROIs were reverse normalized to the space of each subject’s diffusion data according to the following workflow: each subject’s anatomical image (MPRAGE) was normalized to a standard T1-weighted template in MNI space using a symmetric diffeomorphic image registration procedure implemented in the Advanced Normalization Tools (ANTS) Toolbox (Avants et al., 2008). The inverse of this transformation was then applied to all standard space ROIs, bringing each ROI into subject-specific MPRAGE space. Next, each subject’s non-diffusion-weighted b0 reference volume was aligned to their MPRAGE using 12-parameter linear-affine registration using FMRIB’s Linear Image Registration Tool (FLIRT), and the inverse transform of this registration was applied to the ROIs, bringing each ROI into subject-specific diffusion space.

**Figure 1:**
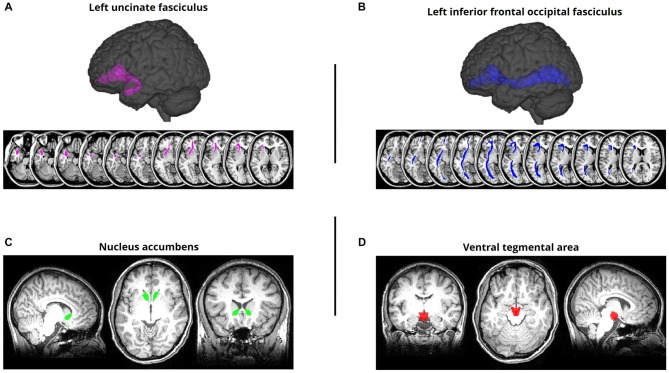
Regions of interest (ROIs). **(A)** Left uncinate fasciculus (UF) overlaid on a standard T1-weighted template in montreal neurological institute (MNI) space. The UF was defined using a probabilistic white matter tractography atlas (Johns Hopkins University [JHU]; Mori et al., 2005). **(B)** Left inferior frontal occipital fasciculus (IFOF) ROI defined using the same procedure. **(C)** Nucleus accumbens (NAcc) ROI, aligned to and overlaid on a representative subject’s MPRAGE. The NAcc was defined using FreeSurfer’s automatic subcortical segmentation routine on the T1-weighted structural image. **(D)** Ventral tegmental area (VTA) ROI, aligned to and overlaid on a representative subject’s MPRAGE. The VTA was defined using a probabilistic atlas of the human VTA (Murty et al., 2014) at a 50% threshold.

ROI masks for tracts of interest were defined based on the Johns Hopkins University (JHU) white matter tractography atlas (Mori et al., 2005,^2^). For each fiber tract, we calculated mean FA values for each individual within separate left hemisphere and right hemisphere ROIs. Our primary fronto-temporal tract of interest were the left and right UF (Figure 1A), following previous work demonstrating the relationship between UF integrity and semantic control (Harvey et al., 2013). We also examined the mean FA of each subject’s left and right IFOF (Figure 1B), as studies have linked this pathway to semantic processing/control (de Zubicaray et al., 2011; Nugiel et al., 2016). As a control analysis, designed to rule out the possibility that generalized differences in white matter tract integrity would correlate with our behavioral measures, we extracted the mean FA of each subject’s left and right corticospinal tract—a tract with no prior association with either reward or memory that has been used as a control pathway in prior studies examining DTI correlations with memory behavior (Winston et al., 2013; Schlichting and Preston, 2016). For all JHU-defined masks, we applied a 10% probability threshold to ensure sufficient coverage of the entire pathway, while avoiding excessive sparsity/shrinkage (that would result if higher thresholds were applied). Since the IFOF and UF masks had considerable anatomical overlap in the JHU atlas, with the UF essentially existing as a subset of the IFOF, we conducted additional analyses in which we excluded all UF voxels from the IFOF mask and only examined the portions of the IFOF that did not show any anatomical overlap with the UF.

For our analysis of anatomical connectivity between the NAcc and VTA, we implemented a probabilistic tractography approach, as no pre-defined atlas was available for this pathway. A left and right NAcc ROI were anatomically defined for each subject using their MPRAGE scan (Figure 1C); this was accomplished using FreeSurfer’s automatic subcortical segmentation routine^3^. Given the challenge of demarcating the anatomical boundaries of the VTA in T1-weighted MR images of individual subjects, we defined a VTA ROI using a probabilistic atlas of human VTA (Murty et al., 2014,^4^) with a 50% probability threshold (Figure 1D).

Using FSL’s PROBTRACKX, in conjunction with BEDPOSTX (Bayesian Estimation of Diffusion Parameters Obtained using Sampling Techniques), each subject’s diffusion image underwent a Bayesian estimation of diffusion parameters at each voxel using a Markov-chain Monte Carlo sampling technique while modeling and accounting for crossing fibers (Behrens et al., 2003, 2007). Using 5000 samples of the distribution of diffusion parameters, 5000 streamlines from each seed voxel were created and this distribution of streamlines was used to create a likely tract location. By taking many such samples, the probabilistic tractography algorithms build up a posterior distribution on the streamline location or the connectivity distribution of each seed ROI to each target ROI.

Our primary measure of interest was the total number of samples from the seed ROI that reached the target mask. To normalize the results and ensure our results would not be driven by variance in the seed ROI size, we divided the total streamline count by the total number of samples sent out from the seed mask (i.e., 5000 * number of voxels in the seed ROI; Johansen-Berg et al., 2005). This tract strength value was then correlated with our behavioral measures of interest. To ensure that our results were not being driven by the size of the target ROI, we computed a partial correlation controlling for the size of the target ROI (note that although the same VTA ROI was used as the target ROI for all subjects, its size varied across participants based on the transformations needed to reverse normalize this ROI from MNI space to the native anatomical space of each subject). Tract strength measures, as indexed by DTI tractography, have been shown to correlate strongly with actual neuroanatomical connectivity as revealed by retrograde tracer injections (Donahue et al., 2016). Because we had *a priori* reason to believe that higher FA values (which reflect increased directional structure of white matter tissue) and higher tract strength values would be an indicator of more robust anatomical connectivity and thus associated with improved task performance, we assessed the significance of the brain-behavior correlations using one-tailed tests. We controlled our false discovery rate (FDR; i.e., Type I error rate) by correcting the observed *p-values* in accordance with the expected proportion of false discoveries amongst the rejected hypotheses for all brain-behavior correlations (Benjamini and Hochberg, 1995). As such, all reported brain-behavior *p-values* have been FDR-corrected, and results that achieve *p* < 0.05 (corrected) are reported as significant. Direct comparisons of a given region’s correlation with two behavioral measures (e.g., high value recall vs. low value recall) were assessed using a two-tailed test for the difference between two dependent correlations with one variable in common (Steiger, 1980) using an online utility (Lee and Preacher, 2013).

## Results

### Behavioral Performance

Our analyses focused on three behavioral measures of interest: (1) *High Value Recall* (the mean number of high value words recalled per list, averaged across the five lists); (2) *Low Value Recall* (the mean number of low value words recalled per list, averaged across the five lists); and (3) *Selectivity Index*. Across participants, the average High Value Recall score was 8.65 (SD = 1.87), which was significantly greater than the average Low Value recall score of 3.18 (SD = 2.72), *t*_(18)_ = 9.27, *p* = 2.84 × 10^–8^. The average Selectivity Index score was 0.605, which was significantly greater than zero (i.e., value-insensitive recall), *t*_(18)_ = 11.48, *p* = 1.03 × 10^–9^.

### Brain-Behavior Correlations: Fractional Anisotropy (FA)

**Figure 2:**
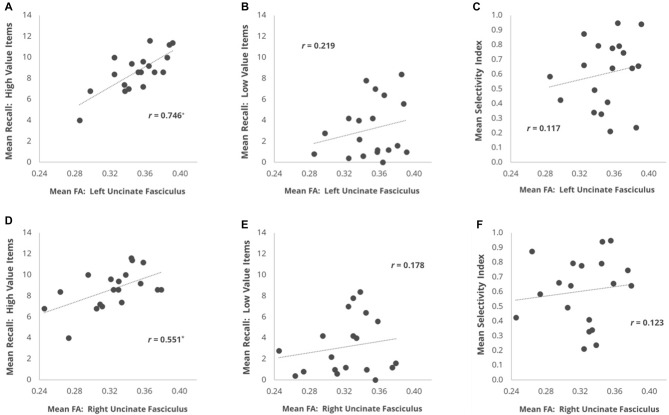
Scatter plots depicting the brain-behavior correlations focused on individual differences in mean fractional anisotropy (FA) within the UF and metrics of memory recall performance. Correlations are plotted for the relationship of mean FA in **(A)** L UF and **(B)** R UF with mean number of high value words recalled. **(C,D)** Same as **(A,B)**, but with mean recall for low value words. **(E,F)** Same as **(A,B)**, but with each subject’s mean Selectivity Index. *p < 0.05 comparing the *r*-value to a one-tailed Student’s *t*-distribution.

We first examined whether individual differences in the mean FA of our primary fronto-temporal pathway of interest, the UF, were correlated with each of our three behavioral measures (Figure 2). For the left UF, we found that mean FA showed a strong positive correlation with High Value Recall (*r* = 0.746, *p* = 0.0025) but not with Low Value Recall (*r* = 0.219, *p* > 0.2), and this difference in correlation magnitude was significant (*z* = 2.606, *p* = 0.0046). For the right UF, we found that mean FA also showed a positive correlation with High Value Recall (*r* = 0.551, *p* = 0.0378) but not with Low Value Recall (*r* = 0.177, *p* > 0.3). However, this difference in correlation magnitude only trended towards significance (*z* = 1.582, *p* = 0.057). A direct comparison between the effects in left and right UF revealed a significantly stronger relationship with High Value Recall performance in the left hemisphere (*z* = 2.099, *p* = 0.018). When correlating mean FA with Selectivity Index, we did not observe a significant effect in either left UF (*r* = 0.177, *p* > 0.3) or right UF (*r* = 0.123, *p* > 0.3).

Mean FA along the IFOF pathway also showed a positive correlation with High Value Recall for both the left (*r* = 0.631, *p* = 0.015) and right (*r* = 0.624, *p* = 0.015) hemisphere ROIs. There was no difference in correlation magnitude as a function of hemisphere (*z* = 0.047, *p* > 0.9). Mean FA in the IFOF did not significantly correlate with Low Value Recall on the left (*r* = 0.308, *p* > 0.2) or right (*r* = 0.336, *p* > 0.1) hemisphere. Despite the finding of significant correlations with High Value Recall and non-significant correlations with Low Value Recall, a direct test of the difference in correlation coefficients failed to yield significant effects in either the left IFOF (*z* = 1.47, *p* = 0.142) or right IFOF (*z* = 1.309, *p* = 0.191). Selectivity Index also showed no relationship with FA in left IFOF (*r* = −0.050, *p* > 0.4) or right IFOF (*r* = 0.047, *p* > 0.3).

Given the strength of our UF findings and the spatial overlap of our atlas-defined UF and IFOF ROIs, we next assessed whether the significant relationship between High Value Recall and FA along the IFOF could potentially be driven by the FA values that were also included in our analyses of the UF. In order to test this hypothesis, we conducted a follow-up analysis where only portions of the left and right IFOF masks that were non-overlapping with the left and right UF masks were analyzed (we refer to resulting ROI as IFOF_exclusive_). We found that mean FA did not significantly correlate with High Value Recall in the left IFOF_exclusive_ (*r* = 0.363, *p* > 0.1) nor Low Value Recall (*r* = 0.177, *p* > 0.2). A similar observation was seen for the right IFOF_exclusive_; mean FA did not significantly correlate with High Value Recall (*r* = 0.417, *p* > 0.1) nor Low Value Recall (*r* = 0.070, *p* > 0.4). When correlating mean FA with Selectivity Index, we did not observe a significant effect in either left IFOF_exclusive_ (*r* = −0.030, *p* > 0.4) or right IFOF_exclusive_ (*r* = 0.170, *p* > 0.2). These results suggest that the value effects documented above for the entire IFOF ROIs were actually driven heavily by FA levels within the anterior portion of these ROIs that overlapped with the UF.

As a control analysis to rule out generic effects of white matter health/integrity and task performance, we examined the mean FA of the corticospinal tract. Mean FA within the left corticospinal tract did not correlate with High Value Recall, Low Value Recall, or Selectivity Index (all *r*’s < 0.238, all *p*’s > 0.1). The same was the case for the right corticospinal tract (all *r*’s < 0.289, all *p*’s > 0.1).

### Brain-Behavior Correlations: Tract Strength

**Figure 3:**
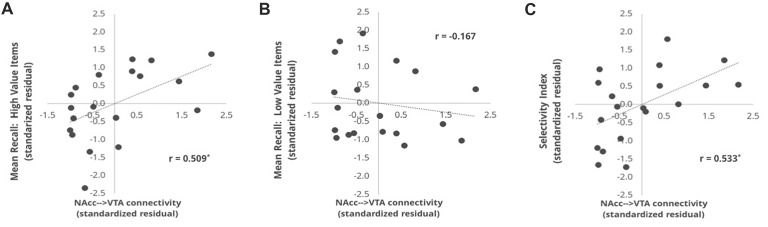
Correlation between NAcc-VTA tract strength and behavioral measures. The tract strength values represent the number of samples that reached the target ROI (VTA) when emanating from a seed ROI (NAcc), using a probabilistic tractography approach and normalizing for the number of samples sent out. The values shown here are standardized residuals controlling for ROI size in each subject. Correlations are plotted for the relationship of NAcc-VTA tract strength and **(A)** the mean number of high value words recalled, **(B)** mean number of low value words recalled and **(C)** Selectivity Index. *p < 0.05 comparing the *r*-value to a one-tailed Student’s *t*-distribution.

Our primary reward circuit pathway of interest was the connection between the NAcc and VTA. Given that our probabilistic VTA ROI was bilateral by nature, we elected to combine the left and right NAcc ROI into a single bilateral NAcc ROI, and we then assessed the relationship between the mean tract strength of the NAcc-VTA pathway and each of our three behavioral performance measures. This was done using partial correlations that controlled for the size of the VTA target ROI, and thus the associated scatterplots (Figure 3) depict the standardized residuals of each variable rather than the raw values. Individual differences in the tract strength of the NAcc-VTA pathway correlated significantly with High Value Recall (*r* = 0.509, *p* = 0.0455) but not with Low Value Recall (*r* = −0.167, *p* > 0.3), and this difference in correlation magnitude was significant (*z* = 2.780, *p* = 0.0054). Furthermore, this pathway’s tract strength correlated significantly with individual differences in Selectivity Index (*r* = 0.533, *p* = 0.0394).

## Discussion

In this study, we used diffusion weighted imaging to assess the relationship between microstructural integrity of white matter pathways and individual differences in value-directed remembering. Our analyses revealed a significant positive correlation between participants’ ability to recall high reward value words and the structural integrity of two white matter pathways of interest: the UF and the tract connecting the NAcc and the VTA. No such correlation was found between these pathways and participants’ recall of low reward value words. Furthermore, the strength of the NAcc→VTA connection was strongly correlated with individual differences in Selectivity Index, suggesting that this mesolimbic pathway may constitute one key determinant of reward-driven modulation of memory encoding behavior.

Prior research using the value-directed remembering paradigm has yielded evidence that participants preferentially engage in deep semantic encoding of high reward value items relative to low reward value items (Castel, 2008; Cohen et al., 2017), and that this is associated with value-related differences in neural activity within lateral prefrontal and temporal lobe regions thought to be key components of the brain’s semantic network (Cohen et al., 2014). Cohen et al. (2016) also found a positive correlation between Selectivity Index and activity in these brain regions during encoding of high reward value items, with no such effect apparent during encoding of low reward value items, suggesting that selectivity in young adults is driven primarily by enhanced semantic encoding of high reward value words.

Motivated by these findings, our DTI analyses focused heavily on exploring whether individual differences in the anatomical robustness of the UF pathway, which connects the ventral PFC with the anterior temporal lobe, might be one factor that predicts memory for high value items. As is common in the DTI literature, we indexed the microstructural integrity of white matter pathways by measuring their mean FA. This measure denotes the degree of restriction that water molecules encounter when diffusing within a given voxel, and as such is increased whenever that voxel’s underlying tissue is rich with coherently oriented myelinated axons. Our finding that the mean FA of participants’ UF predicted their ability to recall high value words, but not low value words, suggests that having a robust UF may be conducive to deploying effective semantic encoding strategies to ensure retention of valuable information. Although this correlation with high value recall was observed in both hemispheres, only in the left UF was the correlation significantly greater with high value recall than low value recall, suggesting that the key behavioral phenomenon in our task—enhanced memory for high value words—may be more strongly associated with fronto-temporal connections within the left hemisphere. This is consistent with our interpretation of this effect as being attributable to the prioritized engagement of semantic processing. We also examined the putative contributions of another major white matter pathway connecting ventrolateral PFC regions with posterior sensory cortices—the IFOF—but found that after excluding the anterior portion of this pathway that overlapped with the UF, its mean FA was uncorrelated with behavioral performance on our task.

In our task paradigm, participants’ ability to remember high value words (i.e., their Mean High Recall score) likely reflects the efficacy with which they can engage in encoding strategies to promote the retention of information they hope to be able to later remember. Early “depth of processing” research demonstrated that elaborative encoding, the process of associating meaning with to-be-remembered information, results in greater retention relative to encoding the information at a superficial level via rote rehearsal (Woodward et al., 1973; Craik and Tulving, 1975; Bradshaw and Anderson, 1982). When tasked with encoding words, those who employ an elaborative encoding strategy are effectively linking the meaning of a word with related concepts—binding its representation into a broader semantic network and creating more potential retrieval routes that could later facilitate successful recall. In this experiment, because some words are deemed to be more valuable to remember than others in regards to the task at hand, it is likely that engagement of elaborative semantic encoding is roughly proportional to the point value assigned each word.

A number of prior studies have linked the UF pathway to aspects of semantic and/or associative encoding. Although our study examines structure-function relationships by capitalizing on individual differences in white matter integrity and behavioral performance in cognitive healthy adults, many valuable insights have been derived from studies of clinical populations or older adults. For instance, in a study of aphasic patients with varying degrees of comprehension deficits, Harvey et al. (2013) found that individual differences in the structural integrity of the left UF were predictive of patients’ performance on tasks requiring semantic control. Specifically, patients with lower UF integrity as indexed by mean FA, showed a diminished ability to ignore semantically related distractors and identify associative relationships when understanding a word. These findings were taken as evidence that the UF plays an important role in semantic control by virtue of its ability to connect cognitive control regions of the anterior ventrolateral PFC with anterior temporal lobe regions thought to be critical for storing word meanings (Visser et al., 2010). Abnormal FA values in the UF have also been correlated with deficits in confrontational naming and semantic memory in patients with temporal lobe epilepsy (McDonald et al., 2008). In further support of the role of UF in semantic processing, studies of semantic dementia patients have frequently reported decreases in FA (or decreases of a related measure known as radial diffusivity) in the UF, particularly in the left hemisphere but occasionally bilaterally (Matsuo et al., 2008; Acosta-Cabronero et al., 2011; Galantucci et al., 2011; Agosta et al., 2012). Individual differences in left UF integrity also correlate with performance on tests of semantic memory in healthy older adults (de Zubicaray et al., 2011). The left UF has also been associated with performance on episodic memory tasks, including the learning of paired associations between visual images (Thomas et al., 2015; Alm et al., 2016) and a task requiring mnemonic control to prioritize the encoding of relative images and ignore distractors (Wendelken et al., 2015). Damage to this pathway is correlated with deficits in immediate and delayed verbal memory (Diehl et al., 2008; McDonald et al., 2008) and visual associative memory (Lockhart et al., 2012).

It is worth noting that not all studies that have examined structural correlates of semantic control have found a reliable correlation with UF integrity. For instance, Nugiel et al. (2016) conducted a verb generation study in which subjects were presented with a noun and asked to generate a related verb. The authors assessed the semantic relatedness between the noun and the provided verb using latent semantic analysis (LSA) and found that individual differences in LSA score (their proxy for semantic control) were not related to FA in the UF, but rather correlated with FA in the left IFOF, and also showed an unanticipated correlation with FA in the inferior longitudinal fasciculus (ILF), a pathway typically associated with high-level vision. While their findings diverge from those of the present study, there were several major methodological differences that may have contributed to this discrepancy. Our atlas-based UF and IFOF ROIs had considerable anatomical overlap in the anterior portion, requiring us to exclusively mask out overlapping voxels to isolate effects that were uniquely attributable to IFOF. As such, our procedure may underestimate the potential contribution of anterior IFOF fibers extending into PFC, whereas Nugiel and colleagues’ use of ROI-to-ROI deterministic tractography may have been more sensitive to these fibers. Furthermore, the tasks used in our respective studies were markedly different, raising the possibility that IFOF integrity is more consequential for the type of semantic control needed to rapidly retrieve word associations, whereas UF integrity may be more important for the type of control needed to facilitate elaborative semantic encoding of words. Future studies will be necessary to better characterize the roles of the UF and IFOF pathways in semantic control and verbal memory.

There is also reason to believe that the UF pathway could more generally play a role in reward-incentivized behavior. For instance, studies in monkeys have shown that the UF is critical for tasks like conditional rule learning where they must associate a particular object with a particular choice location that is rewarded (Parker and Gaffan, 1998; Bussey et al., 2002). In DTI work with human subjects, Camara et al. (2010) found that FA values in a region within the UF correlated with the difference in BOLD activity in the ventral striatum when a participant earned a loss vs. a gain in a gambling task (i.e., was more sensitive to punishments). This finding suggests that the structural integrity of the UF is predictive of an individual’s reward processing behavior. UF FA has also been shown to predict a participant’s ability to delay gratification in a sample of children and adolescents (Olson et al., 2009). These reward-related findings may be attributable to the fact that the UF is a critical pathway connecting parts of the limbic system with the orbitofrontal cortex. Reward contingencies, like those leveraged in our study, have been shown to be encoded in the orbitofrontal cortex (Fellows, 2011), and to depend critically on the integrity of white matter projections from this region (Rudebeck et al., 2013). Given the role of the OFC in maintaining reward representation, it is reasonable to presume that that the OFC would be responsible for relaying that reward information to semantic processing regions within temporal lobe by way of the UF (Olson et al., 2015).

Despite the putative involvement of the UF pathway in reward-driven behavior, we did not find a significant correlation between UF integrity and Selectivity Index—our primary behavioral measure of the degree to which a participant’s encoding efforts were optimized to maximize their accumulation of reward points given the total number of items they were able to recall. To the extent that Selectivity Index can be thought of as a marker of participants’ reward sensitivity, the fact that this measure did not correlate with UF FA suggests that its role in our task paradigm was probably more related to enhancing the encoding of high value items via elaborative semantic encoding rather than adaptively regulating one’s motivation to learn in accordance with item value. That said, individual differences in the Selectivity Index measure did show a significant correlation with the tract strength of a mesolimbic white matter pathway connecting a critical reward-related region of the ventral striatum (NAcc) with a dopamine-producing midbrain region (VTA). In other words, participants with a more robust NAcc-VTA pathway tended to be those individuals who were more selective in their encoding efforts. Selectivity Index increases across lists as participants experience limits in the amount of information that can be recalled on each list (Castel, 2008; Ariel and Castel, 2014). Those participants with stronger anatomical connections in this reward pathway may be more sensitive to feedback on recall performance across lists. These participants may prioritize the encoding of the highest value words given the number of words that can be recalled per list based on task experience—what they learned from performance on prior lists and awareness of their own memory capacity. In this way, mesolimbic reward circuitry may play a key role in the metacognitive ability of adjusting encoding strategy based on experienced recall ability.

Our finding that NAcc-VTA connectivity predicted participants’ selectivity on a value-directed remembering task accords well with prior research linking motivationally significant information to dopaminergic projections from tegmental areas to ventral striatal areas (Camara et al., 2009). Such processes allow for cognitive resources to be geared toward relevant information during memory encoding, as dictated by potential reward (Wittmann et al., 2005, 2008; for review, see Shohamy and Adcock, 2010). In the current study, words preceded by a high value cue are much more indicative of a subsequent reward (i.e., accumulation of points) than their low value counterparts. In our analysis of fMRI data collected from these same participants (Cohen et al., 2014), we found significantly increased activity in both the NAcc and VTA during the encoding of high value vs. low value items. Such engagement of the brain’s core reward circuitry supports the notion that point values, although not linked to monetary gain in our paradigm, were nonetheless processed as salient reward cues and used to modulate behavior (akin to the intrinsic reward value of point accumulation in many video games). The present DTI findings expand upon this result by showing that the robustness of the white matter pathway connecting these two regions is likely one important determinant of both how well, and how selectively, individuals will encode the high value words based on feedback across lists.

Taken together, our results suggest that when presented with a reward value-indicating cue, communication between the NAcc and VTA may act as a gating mechanism to determine if elaborative encoding processes, as facilitated by the UF, will be upregulated to preferentially bolster the encoding of the proceeding word. The UF may fulfill the additional role of facilitating information transmission across the OFC and temporal/limbic regions to continually update the association of a reward value with a word. The integrity of both of these circuits appears to be a critical determinant of behavioral performance in this task paradigm. Although we have been attributing the structural correlates of value-related memory modulation to effects that exert their influence during encoding, it is important to note the possibility that item reward values could impact retrieval dynamics as well. For example, Castel et al. (2013) found that people tend to recall higher value items first, which could be due to the fact that these items were most strongly encoded, but also could be a strategic operation to prevent the buildup of output interference from diminishing the accessibility of high value items. That said, we have reason to believe that the value effects in our study are predominantly indicative of processes engaged at the time of encoding. Post-experiment questionnaires revealed that all participants reported the use of verbal strategies during encoding to help them remember the words (Cohen et al., 2014). Moreover, a series of behavioral experiments using variants of this paradigm found evidence that providing participants feedback on their point totals at the conclusion of each study-test cycle (as was done in the present study) serves to guide learners’ use of metacognitive control to more selectively employ encoding strategies that will promote later recollection of high reward value items (Cohen et al., 2017). Finally, fMRI measurement of brain activity levels during word encoding revealed strong effects of reward value and correlations with Selectivity Index across a number of regions associated with semantic and reward processing (Cohen et al., 2014).

Our findings should be interpreted with some caution given the relatively small size of our sample. Future studies with larger samples would be useful to both assess the replicability of our effects, as well as to explore the putative contributions of additional white matter pathways. Given our limited experimental power, we chose to focus our brain-behavior correlation analyses on a small number of pathways for which the literature provided *a priori* rationale to expect value-related effects. It would also be advantageous for future work to examine the degree to which individual differences in UF and NAcc-VTA integrity predict performance on a wider range of reward-incentive memory tasks. For instance, it is possible that the role of left UF is particularly pronounced for paradigms involving verbal stimuli, for which the use of elaborative semantic encoding strategies is most effective; paradigms using visual stimuli may not show such a structure-function relationship for this pathway. Finally, it will be interesting to explore whether the white matter pathways implicated in our study as predicting value-based memory effects in a sample of younger adults will show similar effects in older adults. Functional neuroimaging work comparing younger and older adults on this paradigm revealed that while both populations show elevated recruitment of the left-lateralized semantic network during the encoding of high value words, younger adults engage these regions—along with reward-related regions—more proactively than older adults (Cohen et al., 2016). Diffusion imaging could offer additional insights into the nature of age-related changes in value-directed remembering and individual differences that predict preserved memory selectivity.

## Author Contributions

NR, MC and JR had full access to all the data in the study and take responsibility for the integrity of the data and the accuracy of the data analysis. MC, AC, BK, NR and JR: study concept and design. NR, MC, ZZ, AC, BK and JR: acquisition, analysis, or interpretation of data; administrative, technical and assessment support. NR and JR: drafting of the manuscript; statistical analysis. BK, MC, JR and AC: obtained funding.

## Conflict of Interest Statement

The authors declare that the research was conducted in the absence of any commercial or financial relationships that could be construed as a potential conflict of interest. The reviewer C-TW and handling Editor declared their shared affiliation.

## Footnotes

^1^http://fsl.fmrib.ox.ac.uk/fsl/fslwiki/FSL

^2^http://cmrm.med.jhmi.edu

^3^http://freesurfer.net/fswiki/SubcorticalSegmentation

^4^http://web.duke.edu/adcocklab
